# Pressure recording analytical method versus PiCCO in hemodynamic unstable patients

**DOI:** 10.1186/cc9483

**Published:** 2011-03-11

**Authors:** A Donati, S Loggi, A Carsetti, MR Lombrano, L Botticelli, A Valentini, V Fiori, R Domizi, C Scorcella, P Pelaia

**Affiliations:** 1 Università Politecnica delle Marche, Ancona, Italy

## Introduction

Hemodynamic monitoring is important for diagnosis and therapy of critically ill patients. Thermodilution is now the gold standard method; however, it cannot be used routinely since it is very invasive. We investigated the agreement between the cardiac index (CI) obtained by mini-invasive monitor MostCare, based on the pressure recording analytical method (PRAM), and by PiCCO thermodilution in hemodynamic unstable patients.

## Methods

We performed a prospective clinical study at our university hospital ICU. Twenty adult patients with hemodynamic instability were enrolled. All patients were sedated and mechanically ventilated with intermittent positive pressure ventilation. The MostCare and PiCCO systems were connected to each patient by a catheter inserted into the femoral artery. For each patient three measurements of CI were simultaneously carried out and the mean was considered for statistical analysis.

## Results

**Figure 1:**
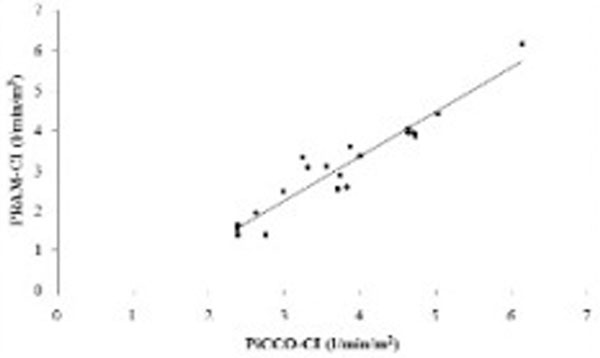
**Linear regression analysis between PRAM-CI and PiCCO-CI**.

**Figure 2:**
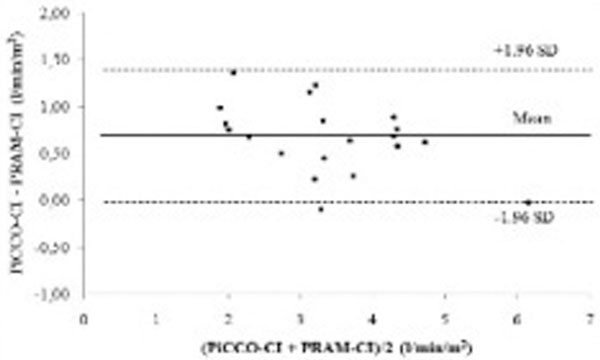
**Bland-Altman plot for comparison between PRAM-CI and thermodilution CI**.

We enrolled 10 severe sepsis/septic shock, four interstitial pneumonia, three COPD, one subarachnoid hemorrhage, one abdominal compartment syndrome, and one polytrauma. The age range was 34 to 84 years (65 ± 13), the APACHE II score range was 13 to 38 (25 ± 6) and SAPS II score range was 22 to 81 (50 ± 16). The correlation coefficient between PRAM-CI and PiCCO-CI was 0.95 (95% CI = 0.89 to 0.99; *P *< 0.001) (Figure 1). The Bland-Altman analysis showed a mean difference between the two methods (bias) of 0.67 ± 0.38 l/minute/m^2 ^with lower and upper 95% limits of confidence of -0.07 and 1.41 l/minute/m^2^, respectively (Figure 2). The percentage of error was 22%.

## Conclusions

This study showed a sufficient agreement between the two techniques. MostCare could be a useful first-level monitoring system, particularly in the first phase of critically ill patients' care or when more invasive systems are not advisable.
